# LSD1 in beige adipocytes protects cardiomyocytes against oxygen and glucose deprivation

**DOI:** 10.22038/IJBMS.2022.65006.14313

**Published:** 2023-01

**Authors:** Yiqiu Cao, Zhu Dong, Dongpeng Yang, Xiaowu Wang

**Affiliations:** 1 Department of Cardiac Surgery, Hainan General Hospital, Hainan Affiliated Hospital of Hainan Medical University, Haikou, 570311, People’s Republic of China; 2 The First School of Clinical Medicine, Southern Medical University, Guangzhou, 510515, People’s Republic of China; 3 Department of Cardiovascular Surgery, Zhujiang Hospital, Southern Medical University, Guangzhou, 510280, People’s Republic of China; 4 Department of Cardiovascular Surgery, Guangzhou Red Cross Hospital, Jinan University, 510235, People’s Republic of China; # These authors contributed eqully to this work

**Keywords:** Beige adipocyte, Cardiomyocyte, Epicardial adipose tissue, Lysine-specific demethylase 1, Oxygen and glucose-deprivation

## Abstract

**Objective(s):**

Epicardial adipose tissue (EpAT) is known for its role in supporting the cardiomyocytes. Lysine-specific demethylase 1 (LSD1), a typical lysine demethylase, is an essential regulator for the maintenance of beige adipocytes. However, the effect of LSD1 in the adipogenic differentiation of beige adipocytes in EpAT, and its function on oxygen and glucose deprivation (OGD)-injured cardiomyocytes remain unclear.

**Materials and Methods:**

Heart tissues from young mice and elder mice were collected for immunohistochemical staining. LSD1 in 3T3-L1 cells was knocked down by LSD1-shRNA lentivirus infection. The qRT-PCR, western blotting, and Oil Red O staining were employed to detect the adipogenic differentiation of 3T3-L1 cells and formation of beige adipocytes. The cardiomyocytes co-cultured with beige adipocytes were used for OGD treatment. Cell apoptosis was analyzed by flow cytometry. The lactate dehydrogenase (LDH) and superoxide dismutase (SOD) activity were analyzed using commercially available kits.

**Results:**

The decrease of LSD1 was related to the age-dependent loss of beige adipocytes in mice EpAT. LSD1 knockdown inhibited the adipogenic differentiation of 3T3-L1 cells and formation of beige adipocytes. The down-regulation of LSD1 in 3T3-L1 cells decreased the protective effect of mature adipocytes on OGD-injured cardiomyocytes.

**Conclusion:**

The decreased expression of LSD1 in mice EpAT was associated with age-dependent ablation of beige adipocytes. The protective effect of beige adipocytes on OGD-injured cardiomyocytes is reduced by knockdown of LSD1 in adipocytes. The present study provided exciting insights into establishing novel therapies against age-dependent cardiac diseases.

## Introduction

High mortality due to heart failure is a common health problem all over the world. The epicardial adipose tissue (EpAT), located primarily around cardiac blood vessels in the atrioventricular cleft and around the right ventricle, is a rich source of adipokines (1). By interacting with cardiac myocytes and other tissues in a paracrine manner, EpAT promotes efficient cardiac functions (2). Besides, EpAT ensures thermal and physical protection for the heart by supporting the heart muscle (3-5). 

EpAT is a dynamic fat depot consisting of white adipocytes, preadipocytes, and stroma-vascular cells as well as ganglionic, nerve, and immune cells. Epicardial adipocytes can increase or remodel in pathological conditions, such as obesity and inflammation *in vivo* (6, 7). White adipocytes, primarily serving lipid storage, contain a large lipid droplet and few mitochondria. Adrenergic stimuli or cold can induce the white adipose tissue into uncoupling protein-1 (UCP1)-positive brown-like adipocytes, termed beige adipocytes (8, 9). Similar to brown adipocytes, beige adipocytes also contain multiple smaller (multilocular) lipid droplets and abundant mitochondria. UCP1, a mitochondrial membrane protein highly expressed in brown adipocytes and beige adipocytes, provides these cells with the unique ability to dissipate large quantities of energy as heat (10). Multilocular UCP1-positive beige adipocytes are lost from white adipose tissue depots with the aging of mice and are replaced with white adipocytes (11).

Lysine-specific demethylase 1 (LSD1), a typical lysine demethylase (12), regulates epigenetic modification crucial to many physiological processes, such as cell growth, differentiation, and metabolism (13). LSD1 was required for differentiation and function of adipocytes (14). It was shown that LSD1 levels were decreased during aging, concomitantly with a transition of beige into white adipocytes and a loss of thermogenic properties (14). LSD1 overexpression prevents the age-related transition of beige adipocytes to white adipocytes (15). Peroxisome proliferator-activated receptor α (PPARα) was involved in the function of LSD1, and activation of PPARα with an agonist rescued the loss of beige adipocytes in LSD1-KO mice (14, 15). Therefore, LSD1 is an essential regulator for the maintenance of beige adipocytes. However, the function of LSD1 expressed in adipocytes on cardiomyocytes was still limited. 

In this study, we aimed to explore the role of LSD1 expressed on EpAT in oxygen and glucose deprivation (OGD) injured cardiomyocytes.

## Materials and Methods

***Animals***

C57BL/6J male mice were purchased from Chengdu Dossy Experimental Animals Co., Ltd (Chengdu, China). All mice were maintained in a ventilated chamber, and food and water were available *ad libitum*. All animal protocols were approved by the Laboratory Animal Care and Use Committee at Southern Medical University, Guangzhou, China (No. 201638, 2016).

***HE and immunohistochemical staining***

The heart tissues from young mice (6 months) and elder mice (20 months) were collected and fixed in paraformaldehyde, embedded in paraffin, serially sectioned, and stained with hematoxylin-eosin (HE). The immunohistochemical staining of the paraffin sections was performed according to the standard procedures. The sections of heart tissue from mice were incubated with DLK-1 (ab209483, 1: 200, Abcam, Cambridge, UK), PPARγ (ab209350, 1: 200, Abcam), and UCP1 antibodies (ab209483, 1: 500, Abcam) at 4 ^°^C overnight. After incubation with HRP-labeled goat anti-rabbit IgG (ab97080, 1: 10000, Abcam) for 1 hr, the sections were stained with diaminobenzidine and counterstained with hematoxylin. The images were captured using a microscope (Leica DM4000 B LED, Leica Microsystems, Heidelberg, Germany).

***Immunofluorescence analysis***

The 5 μm paraffin sections of heart tissues from the young and elder mice were deparaffinized and rehydrated, and antigen retrieval was performed by boiling for 20 min with a citrate buffer. The sections were blocked with 5% goat serum (Beyotime, Shanghai, China) for 1 hr and incubated with Alexa Fluor® 488 conjugated LSD1 antibody (sc-271720AF488, 1: 100, Santa Cruz Biotechnology, Santa Cruz, CA, USA) and Alexa Fluor® 594 conjugated UCP1 antibody (ab225490, 1: 100, Abcam) for 2 hr at room temperature in the dark. Images were captured using a laser confocal microscope (LSM 900 with Airyscan 2, ZEISS, Shanghai, China). 

***Total antioxidant capacity (TAC) assay***

Blood samples were collected from young mice (6 months) and elder mice (20 months). Then the serum was extracted from the blood sample by centrifugation at 1000 × g for 15 min. Total antioxidant capacity (TAC) in the serum was detected using total antioxidant capacity assay (ferric reducing antioxidant power method) commercial kits (Nanjing Jiancheng Bioengineering Institute, Nanjing, China). In this method, Fe^3+^-TPTZ is reduced to Fe^2+^-TPTZ by antioxidants present in the sample. The absorption at the wavelength of 593 nm was read using a spectrophotometer. The TAC of the samples was calculated based on the standard curve. 

***Creatine kinase isoenzyme (CK-MB) detection***

The CK-MB levels in the serum of mice were detected using a creatine kinase MB isoenzyme assay kit (immunoinhibition method) purchased from Nanjing Jiancheng Bioengineering Institute (Nanjing, China). 

***Cell culture and adipogenic induction***

The preadipocytes, murine 3T3-L1 cells, were cultured with high glucose DMEM (Gibco, Carlsbad, CA, USA) supplemented with 10% fetal bovine serum (FBS, Gibco) and 1% penicillin-streptomycin at 37 ^°^C and 5% CO_2_ incubator. 

Beige adipocyte induction was performed according to previous reports (16). Briefly, 3T3-L1 preadipocyte cells were seeded in 6-well plates (4×10^5^ cells/well) until reaching 70%–80%. The medium was replaced with DMEM supplemented with a differentiation cocktail containing 0.5 mM of 3-isobutyl-1-methylxanthine (IBMX, Sigma, St. Louis, MO, USA), 0.25 μM of dexamethasone (Sigma) and 10 μg/ml of insulin (Sigma). After 48 hr, the differentiation cocktail was removed and the medium was replaced by DMEM supplemented with triiodothyronine (50 nM, Selleck, Shanghai, China), rosiglitazone (1 µM, Sigma), and IBMX (0.5 mM) for beige adipocytes differentiation. The medium was renewed every 2 days. After a 14 day induction, the cells were collected for western blotting assay, oil red O staining assay, or co-culturing with cardiomyocytes.

***Cell infection***

For the virus infection, 3T3-L1 cells (4×10^4^ cells/well) were seeded into 6-well plates in a growth medium. The next day, the culture medium was replaced with fresh medium and the 3T3-L1 cells were infected with the LSD1-shRNA lentivirus (LV-LSD1-shRNA1, LV-LSD1-shRNA2, LV-LSD1-shRNA3 or LV-LSD1-shRNA4) or negative-shRNA lentivirus (Hunan Fenghui Biotechnology Co., Ltd., Changsha, China). The lentivirus-containing medium was replaced with fresh growth medium 24 hr after infection, and the fluorescence intensity was observed under a fluorescent inverted microscope (Olympus, Beijing, China) 48 hr after infection. The LV-LSD1-shRNA infected 3T3-L1 cells were screened with 5 μM of puromycin (A1113803, ThermoFisher, Waltham, MA, USA) for a week to generate LSD1 stable knockdown 3T3-L1 cell lines for subsequent differentiation assay.

***Quantitative real-time polymerase chain reaction (qRT-PCR)***

LSD1 down-regulated 3T3-L1 cells were collected, and TRIzol was added to extract total RNA. According to the instructions of the PrimeScript RT Master Mix (TaKaRa, Dalian, China), 1 μg RNA was reversely transcribed into the cDNA. The mRNA levels of the LSD1 gene were measured using SYBR^®^ Premix Ex Taq^TM ^II (QIAGEN, Shanghai, China) according to the manufacturer’s instructions. The primers for qRT-PCR were designed by Primer Premier 5: LSD1: 5’-CCCAAGGAA ACTGTGGTATC-3’ (sense) and 5’-GATCGGCTGAGCCATTAA A-3’ (antisense); β-actin: 5’-ACAGAGCCTCGCCTTTGC-3’ (sense) and 5’-GCGGCGATATCATCATCC-3’ (antisense). All of the primers were synthesized by Sangon Biotech Co., Ltd. (Shanghai, China). The β-actin was used as an internal reference. Relative gene expression levels were calculated using the 2^−ΔΔCt^ method.

***Western blotting***

The differentiated adipocytes were lysed using RIPA buffer (P0013C, Beyotime, Shanghai, China) at 4 ^°^C. The lysate was centrifuged at 12 000 rpm for 10 min, and the protein concentration was determined with the Bradford Protein Assay Kit (P0006, Beyotime). Twenty micrograms of total protein were subjected to 10% SDS-PAGE. The protein in the gel was transferred to the PVDF membrane. After blocking, the membrane was incubated with the following primary antibodies overnight at 4 ^°^C: LSD1 antibody (ab129195, 1: 1000, Abcam), delta-like 1 protein (DLK-1) antibody (ab210471, 1: 1000, Abcam), peroxisome proliferator-activated receptor γ (PPARγ) antibody (ab209350, 1: 1000, Abcam), UCP1 antibody (ab209483, 1: 1000, Abcam), CCAAT-enhancer-binding protein α (C/EBPα) antibody (ab40764, 1: 1000, Abcam), CD137 antibody (ab243648, 1: 1000, Abcam), or β-actin antibody (ab8227), 1: 5000, Abcam). Positive signals were detected after incubation with corresponding HRP-labeled goat anti-rabbit IgG (ab97080, 1: 10000, Abcam) for 1 hr at room temperature. Protein bands were detected using the BeyoECL Moon kit (Beyotime), and the intensity was quantified using the ImageJ 1.47 software.

***Oil Red O staining***

The differentiated adipocytes were rinsed twice with phosphate buffer saline (PBS) and fixed with 4% paraformaldehyde for 15 min at room temperature. After washing twice with PBS, the cells were stained with 0.6% Oil Red O dye for 30 min and rinsed with 60% ethanol. Finally, the cells were washed twice with distilled water and photographed.

***Isolation of primary cardiomyocyte***

The hearts were collected from the neonatal C57 BL/6J male mice, washed, and squeezed in frozen PBS to remove the blood and other tissues. Then, the heart tissues were cut into 1 mm^3^ blocks and digested in a 10 ml enzyme mixture containing 0.5 mg/ml collagenase II and trypsin (Gibco) in PBS. The digestion was terminated with DMEM (high glucose) containing 10% FBS and the cell suspension was filtered through a 40 μm cell strainer. After centrifugation at 1000 rpm for 5 min, the supernatant was discarded and the cells were washed with PBS thrice. After 2 hr of differential adhesion, the obtained primary cardiomyocytes were cultured in DMEM (high glucose) containing 10% FBS. 

***Co-culture and oxygen and glucose deprivation (OGD) treatment***

The co-culture experiment of cardiomyocytes with differentiated adipocytes was performed using transwell chambers (0.4 μm diameter pore, Corning) to avoid direct contact between 3T3-L1 adipocytes and cardiomyocytes as previously described (17, 18). Briefly, primary cardiomyocytes (0.5×10^6^ cells/well) were seeded in the upper chamber and cultured in DMEM (high glucose) containing 10% FBS for 24 hr before co-culture. Before co-culture, both beige adipocytes differentiated from 3T3-L1 cells (1×10^6^ cells/well in 6-well plates) and cardiomyocytes were washed with PBS. All co-culture experiments were done in serum-free DMEM at 37 ^°^C and 5% CO_2_ incubator. 

The cardiomyocytes co-cultured with beige adipocytes for 24 hr were used for OGD treatment. Briefly, the medium was replaced with glucose-free DMEM. The cells were incubated in a three-gas incubator with 1% O_2_, 5% CO_2_, and 94% N_2_, at 37 ^°^C for 12 hr (19). Then the lactate dehydrogenase (LDH) and superoxide dismutase (SOD) activity in the supernatant of each well were detected using commercially available kits (C0016 and S0109, Beyotime) according to the manufacturer’s instructions. The cardiomyocytes in the upper chamber were harvested for cell apoptosis analysis. 

***Cell apoptosis analysis***

The cardiomyocytes co-cultured with beige adipocytes were washed with PBS and fixed with 70% ethanol, followed by double-staining with Annexin V-FITC and propidium iodide (PI). Then, flow cytometry analysis was performed with a fluorescence-activated cell sorter (FACS; CytoFLEX, American, Beckman Coulter), and the data were analyzed using the FCS Express 5 software.

***Statistical analysis***

The data were expressed as mean±SD. The statistical analysis was conducted using GraphPad Prism 7.0. Comparisons among multiple groups were calculated using one-way ANOVA with a Bonferroni assay. *P*<0.05 indicated a significant difference.

## Results

***The decreased LSD1 was related to the age-dependent loss of beige adipocytes in mice EpAT***

The concentration of serum creatine kinase isoenzyme MB (CK-MB) is generally used as one of the indicators for myocardial injury (20). In this study, we detected CK-MB in the serum of young mice (6 months) and elder mice (20 months). The data showed the serum levels of CK-MB were slightly higher in the elder mice than those in the young mice, but the difference was not significant (Table 1). Literature data showed that total antioxidant capacity (TAC) decreased significantly with age (21). Here, we also showed that the serum levels of TAC were significantly decreased in the elder mice, as compared with the young mice (Table 1). 

**Figure 1:**
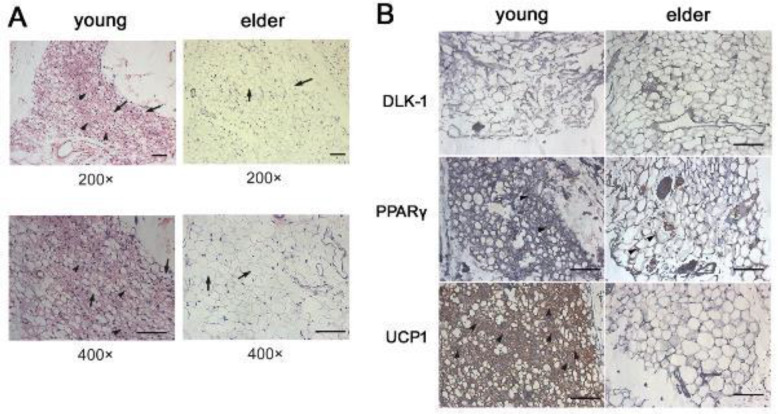
Morphology changes of Epicardial adipose tissue (EpAT) in young and elder mice

To investigate whether beige adipocytes in EpAT decreased in the aging, the EpAT from young mice (6 months) and elder mice (20 months) were collected. HE staining exhibited that the EpAT from young mice consisted of white adipocytes (characterized by unilocular large lipid droplets) and beige adipocytes (characterized by multiple small lipid droplets). In contrast, the EpAT from elder mice was mainly composed of white adipocytes (Figure 1A). These data indicated that beige adipocytes in elder mice EpAT were decreased. 

The delta-like 1 protein (DLK-1, also known as preadipocyte factor 1) is specifically expressed in preadipocytes but not in adipocytes and thus is used as a preadipocyte marker (22). The peroxisome proliferator-associated receptor γ (PPAR γ) is associated with adipocyte cell differentiation and can be used as a marker of mature adipocytes (23). UCP1 was specifically expressed on brown adipocytes and beige adipocytes (24). So, the expression of DLK-1, PPARγ, and UCP1 in the EpATs from young and elder mice was detected by immunohistochemical staining. The data showed there was no significant difference in DLK-1 and PPARγ expression between EpATs from young and elder mice (Figure 1B), suggesting that preadipocytes and mature adipocytes in EpAT presented no obvious change between young and elder mice. The EpAT from young mice contained their pool of UCP1-positive beige adipocytes which was absent in elder mice (Figure 1B). 

**Figure 2:**
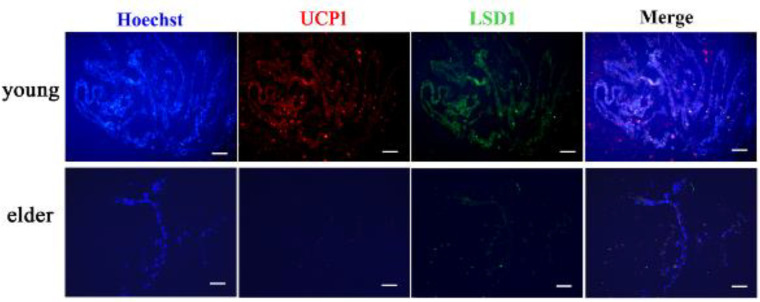
Co-localization of uncoupling protein-1 (UCP1) positive beige fat cells and lysine-specific demethylase 1 (LSD1) in the EpAT of young and elder mice

A previous study demonstrated that the expression of LSD1 was decreased in aging inguinal white adipose (15). In the present study, immunofluorescence assay confirmed that UCP1 and LSD1 expression were both decreased in heart tissue sections from elder mice, as compared with that from the young mice (Figure 2). These data indicated that LSD1 might be related to the reduction of UCP1-positive beige adipocytes in elder mice. 

***LSD1 participated in the formation of UCP1-positive beige adipocytes***

**Figure 3:**
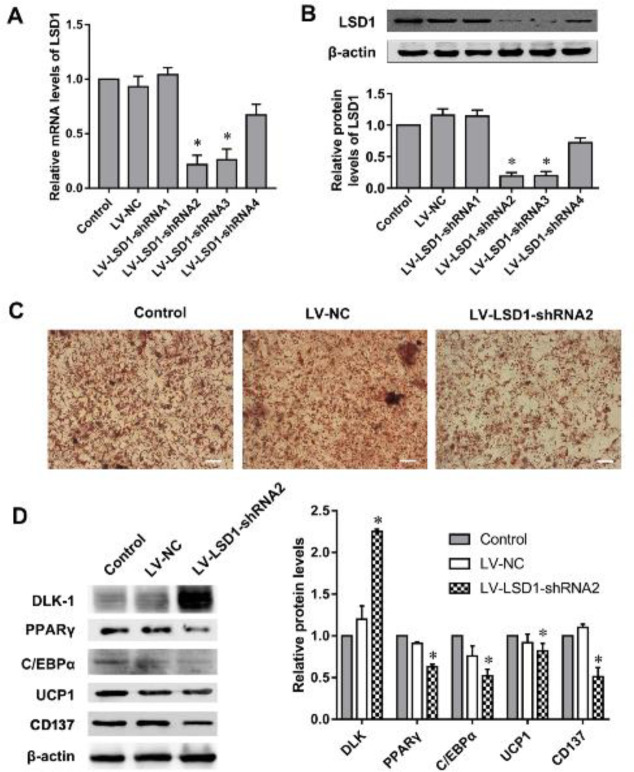
Down-regulation of lysine-specific demethylase 1 (LSD1) in 3T3-L1 cells is associated with reduced formation of uncoupling protein-1 (UCP1) positive brown-like adipocytes

In order to study the function of LSD1 in beige adipocyte induction, 3T3-L1 cells were infected with LSD1-shRNA lentivirus. The mRNA and protein levels of LSD1 in the LV-LSD1-shRNA-2 or LV-LSD1-shRNA-3 infected cells were significantly decreased compared with the LV-NC group (Figures 3A and B). LV-LSD1-shRNA-2 was thus selected for LSD1 knockdown in the following experiments. Oil red O staining revealed that the LSD1 down-regulation markedly inhibited lipid accumulation, especially decreasing the size of lipid droplets during adipogenic differentiation (Figure 3C). After adipogenic induction for 14 days, the protein levels of adipogenic differentiation-related transcription factors were detected. The data showed that protein expression of DLK-1 (marker of preadipocytes) was significantly increased, while the protein expression of PPARγ and C/EBPα (marker of mature adipocytes) was markedly decreased in the LSD1 down-regulated 3T3-L1 cells (Figure 3D). The expression of UCP1 and CD137, beige fat-specific markers (25), was also significantly decreased in LSD1 down-regulated 3T3-L1 cells (Figure 3D). These data suggested that LSD1 knockdown inhibited the adipogenic differentiation.

***LSD1 knockdown decreased the protective effect of beige adipocytes on OGD-injured cardiomyocytes***

**Figure 4:**
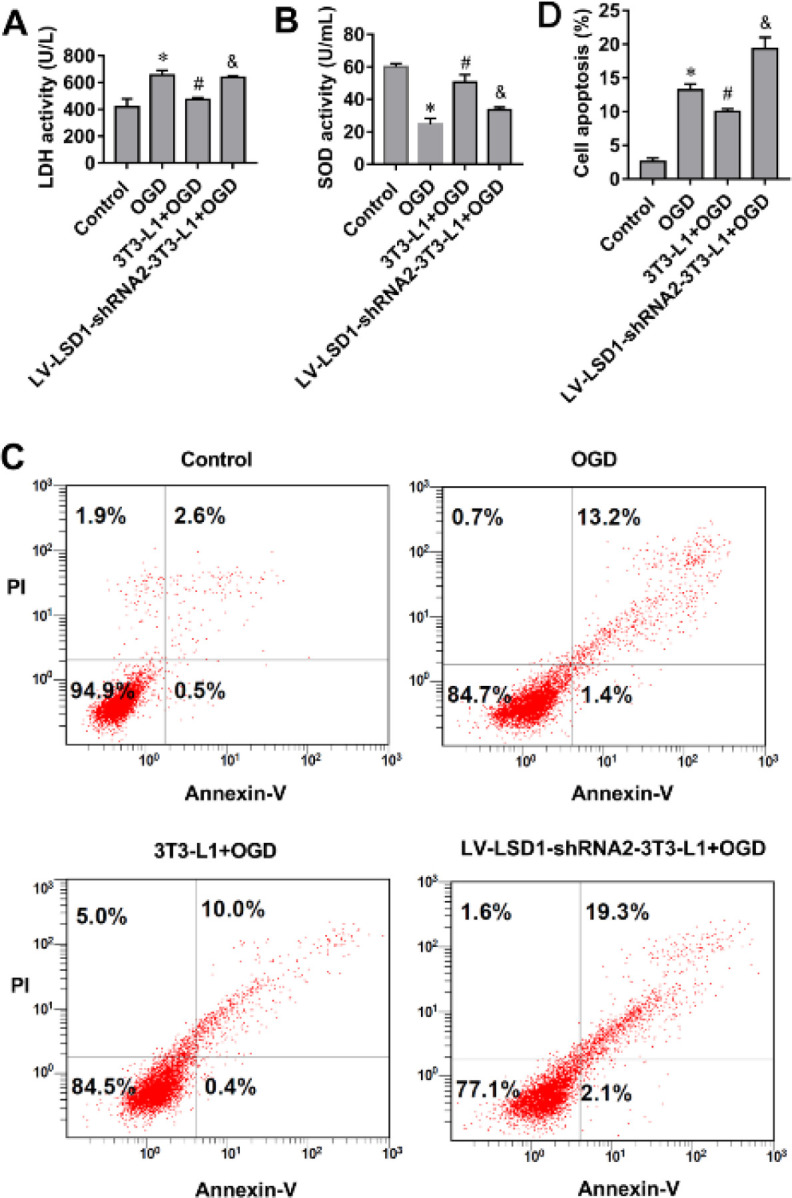
LSD1 knockdown decreases the protective effect of mature adipocytes on oxygen and glucose deprivation (OGD)-injured cardiomyocytes

It was reported that LSD1 was essential for the formation of UCP1-positive brown-like adipocytes and adipogenic differentiation of 3T3-L1 cells (26). To investigate whether LSD1 knockdown in 3T3-L1 cells affects OGD-injured cardiomyocytes, we co-cultured cardiomyocytes with 3T3-L1 cells and analyzed SOD activity, LDH activity, and cell apoptosis of cardiomyocytes. The results showed that OGD treatment increased LDH release and cell apoptosis rate, and decreased SOD activity in cardiomyocytes (Figure 4). Co-culture with adipocytes derived from normal 3T3-L1 cells could reverse OGD-induced increase of LDH release and cell apoptosis of cardiomyocytes (Figure 4). SOD activity in OGD-treated cardiomyocytes was also increased upon co-culture with adipocytes derived from normal 3T3-L1 cells (Figure 4B). However, LDH release and apoptosis rate were not significantly reduced in cardiomyocytes co-cultured with adipocytes derived from LSD1-down-regulated 3T3-L1, as compared with the OGD group (Figure 4). SOD activity in cardiomyocytes co-cultured with adipocytes derived from LSD1-down-regulated 3T3-L1 was not significantly increased, as compared with the OGD group (Figure 4B). These data suggested that LSD1 knockdown restrained the protective effect of mature adipocytes on OGD-injured cardiomyocytes. 

## Discussion

The formation and maintenance of beige adipocytes provide exciting insights into establishing novel approaches to prevent heart diseases. Despite the broad interest in beige adipocytes sparked in recent years, the process of progressive age-related beige-to-white transition and the effect of beige adipocytes on cardiomyocytes remains unclear. In this study, we identified the expression changes of LSD1 in the EpAT of aging mice. Furthermore, LSD1 affected the differentiation of 3T3-L1 cells as well as the formation of beige adipocytes, which played a protective role on cardiac cardiomyocytes upon OGD-evoked injury.

EpAT, with adipose tissue penetrating the heart muscle, defines the close anatomic relationship with the myocardium and secretes a wide range of adipocytokines affecting myocardial biology (27). The functional relationship between the heart and the overlying EpAT is important. Indeed, the adipocytes within EpAT and their products are critical to heart function. EpAT provides thermal and physical protection for the heart while beige adipocytes in EpAT are thermogenic competent, implying its role in protecting the heart against hypothermia (3, 14, 28). Aging is accompanied by dramatic metabolic changes, with beige adipocytes decreasing and white adipocytes accumulating in EpAT. LSD1 plays a role in the age-dependent loss of beige fat cells (15). Duteil *et al*. (15) found that LSD1 levels decreased with age in inguinal white adipose tissue concomitantly with the age-related loss of beige adipocytes. Consistent with the previous study, we found that the beige adipocytes were absent in aging mice. We also found that LSD1 is co-localized with UCP1-positive cells in mice EpAT, suggesting that LSD1 might contribute to the changes of UCP1-positive adipocytes. Related studies presented that LSD1 is essential for the maintenance of beige adipocytes through PPARα. The PPARα agonist GW9578 positively regulates the expression of UCP1 in brown adipocytes, and the pharmacological activation of PPARα rescues the loss of beige adipocytes in LSD1-KO mice (15, 29). In the present study, we further identify that LSD1 is essential for the formulation of beige adipocytes (UCP1-positive brown adipocytes) in EpAT. LSD1 down-regulation in 3T3-L1 cells inhibited the formation of UCP1-expressing beige adipocytes. LSD1 is also an epigenetic modifier involved in the early commitment of preadipocytes (14). Musri *et al*. (30) found knock-down of LSD1 impairs the adipogenic differentiation of 3T3-L1 preadipocytes, suggesting a role of LSD1 in adipogenesis. Consistent with previous studies, our results confirmed that LSD1 down-regulation inhibited the adipogenic differentiation of 3T3-L1 cells. To further verify the function of LSD1 on beige adipocytes, the 3T3-L1 cells were infected with LSD1 overexpression lentivirus. Unfortunately, we found that LSD1 overexpression led to cell death (Figure S1). The molecular mechanisms need further investigation.

Adipose tissue is an important organ for energy homeostasis and EpAT plays a significant role, as the excess of cardiac fat can contribute to the progression of heart disease (6). Excessive accumulation of EpAT is associated with atrial fibrillation (AF) development (31). The adipose tissues in the excessive accumulation of EpAT have pro-arrhythmia effects (32, 33). Previous studies have explored that obesity-associated cardiovascular diseases can be treated through stimulation of white adipocytes to transform into beige adipocytes *in vivo* (34, 35). Thus the various types of adipocytes in EpAT are relevant to cardiac health. Beige adipocytes possess the remarkable capacity to dissipate energy via UCP1 (36, 37). In this study, we found that LSD1 down-regulation inhibits the differentiation from preadipocytes to beige adipocytes, affecting the protective role of EpAT. In contrast, induced differentiation in beige adipocytes can fight against OGD injury on co-cultured cardiomyocytes. We suggested that beige adipocyte induction would be a feasible approach to protect cardiomyocytes in OGD injury. Several studies present that a wide range of adipokines secreted by EpAT can also protect the heart against oxidative stress in the myocardium and regulate cardiac function (38, 39). During myocardial ischemia/reperfusion, oxidative stress was the main factor leading to ischemia/reperfusion injury (40-42). Under conditions of increased myocardial oxidative stress, the heart releases transferable mediators, such as 4-hydroxynonenal (4HNE), which act as signaling molecules to up-regulate adiponectin expression of adipokines in EpAT in a PPARγ–dependent manner (27). The up-regulated adiponectin in EpAT represents a protective paracrine response of EpAT against myocardial oxidative stress. EpAT-released orosomucoid inhibits caspase-3-mediated apoptosis of cardiomyocytes (7). In this study, we also found that UCP1-expressing adipocytes differentiated from 3T3-L1 cells significantly alleviated the apoptosis of co-cultured cardiomyocytes in OGD conditions. 

**Table 1:** Creatine kinase isoenzyme (CK-MB) and Total antioxidant capacity (TAC) levels in the serum of young and elder mice (n=8)

	Creatine kinase-MB (U/L)	Total antioxidant capacity (μmol/L)
Young mice	41.24 ± 4.31	1254.00 ± 104.80
Elder mice	54.31 ± 6.37	812.30 ± 68.62*
*t*	1.700	3.523
*P*	0.111	0.003

Variables are shown as the mean±SD. *P<0.05

(A) Hematoxylin and eosin (HE) staining of mice EpAT. The arrows indicate white adipocytes, characterized by unilocular large lipid droplets. And the arrowheads indicate beige adipocytes, characterized by multiple small lipid droplets. (B) Immunohistochemical staining of DLK-1, PPARγ, and uncoupling protein-1 (UCP1) in the EpAT of mice. The arrowheads indicate positive expression. n=6. Bar scale, 40 μm

The expression of UCP1 and LSD1 in EpAT of young and elder mice was detected by immunofluorescence. n=6. Bar scale, 40 μm

3T3-L1 cells were infected with LSD1-shRNA lentivirus. The mRNA (A) and protein (B) expression of LSD1 was detected by RT-PCR and western blotting, respectively. (C) Oil red O staining of differentiated 3T3-L1 cells infected with or without LV-LSD1-shRNA. (D) Protein level of adipogenic differentiation markers was determined using Western blotting. Bar scale, 40 μm, *P<0.05 vs LV-NC group

Control group: primary cardiomyocytes were cultured in normal conditions. OGD group: primary cardiomyocytes were cultured under ODG conditions. 3T3-L1+OGD group: 3T3-L1 cells were induced to adipogenic differentiation and co-cultured with OGD-injured primary cardiomyocytes. LV-LSD1-shRNA2-3T3-L1+OGD group: lysine-specific demethylase 1 (LSD1)-shRNA lentivirus infected 3T3-L1 cells were induced to adipogenic differentiation and co-cultured with OGD-injured primary cardiomyocytes. (A) lactate dehydrogenase (LDH) production in cardiomyocytes was detected by the LDH assay kit. (B) superoxide dismutase (SOD) activity in cardiomyocytes was detected by the SOD assay kit. (C) Cell apoptosis of cardiomyocytes was detected by flow cytometry analysis. (D) Quantitative data of cell apoptosis of cardiomyocytes was detected by flow cytometry analysis

*P<0.05 vs control group, ^#^*P<*0.05 vs OGD group, ^&^*P<*0.05 vs 3T3-L1+OGD group

## Conclusion

This study demonstrated that LSD1 was related to the age-dependent depletion of beige adipocytes and contributed to the formation of beige adipocytes. LSD1 was essential for the protective role of beige adipocytes on OGD-injured cardiomyocytes. The present study provided exciting insights into establishing novel therapies against age-dependent cardiac diseases**. **

## Ethical approval

All animal protocols were approved by the Laboratory Animal Care and Use Committee at Southern Medical University, Guangzhou (No. 201638, 2016).

## Authors’ Contributions

CYQ and WXW designed the study. CYQ, DZ, and YDD performed the experiments and analyzed the data. WXW administrated the project. CYQ, DZ, and WXW drafted the original manuscript. All authors read and approved the final manuscript.

## Conflicts of Interest

The authors declare no conflicts of interest.
